# From Where Are Tuberculosis Patients Accessing Treatment in India? Results from a Cross-Sectional Community Based Survey of 30 Districts

**DOI:** 10.1371/journal.pone.0024160

**Published:** 2011-09-02

**Authors:** Srinath Satyanarayana, Sreenivas Achutan Nair, Sarabjit Singh Chadha, Roopa Shivashankar, Geetanjali Sharma, Subhash Yadav, Subrat Mohanty, Vishnuvardhan Kamineni, Nevin Charles Wilson, Anthony David Harries, Puneet Kumar Dewan

**Affiliations:** 1 International Union against Tuberculosis and Lung Disease (The Union), South-East Asia Regional Office, New Delhi, India; 2 Center for Operations Research, The Union, Paris, France; 3 Center for Chronic Disease Control (CCDC), New Delhi, India; 4 London School of Hygiene and Tropical Medicine, London, United Kingdom; 5 World Health Organization, India Country Office, New Delhi, India; McGill University, Canada

## Abstract

**Background:**

Tuberculosis (TB) notification in India by the Revised National TB Control Programme (RNTCP) provides information on TB patients registered for treatment from the programme. There is limited information about the proportion of patients treated for TB outside RNTCP and where these patients access their treatment.

**Objectives:**

To estimate the proportion of patients accessing TB treatment outside the RNTCP and to identify their basic demographic characteristics.

**Methods:**

A cross sectional community-based survey in 30 districts. Patients were identified through a door-to-door survey and interviewed using a semi-structured questionnaire.

**Results:**

Of the estimated 75,000 households enumerated, 73,249 households (97.6%) were visited. Of the 371,174 household members, 761 TB patients were identified (∼205 cases per 100,000 populations). Data were collected from 609 (80%) TB patients of which 331 [54% (95% CI: 42–66%)] were determined to be taking treatment ‘under DOTS/RNTCP’. The remaining 278 [46% (95% CI: 34–57%)] were on treatment from ‘outside DOTS/RNTCP’ sources and hence were unlikely to be part of the TB notification system. Patients who were accessing treatment from ‘outside DOTS/RNTCP’ were more likely to be patients from rural areas [adjusted Odds Ratio (aOR) 2.5, 95% CI (1.2–5.3)] and whose TB was diagnosed in a non-government health facility (aOR 14.0, 95% CI 7.9–24.9).

**Conclusions:**

This community-based survey found that nearly half of self-reported TB patients were missed by TB notification system in these districts. The study highlights the need for 1) Reviewing and revising the scope of the TB notification system, 2) Strengthening and monitoring health care delivery systems with periodic assessment of the reach and utilisation of the RNTCP services especially among rural communities, 3) Advocacy, communication and social mobilisation activities focused at rural communities with low household incomes and 4) Inclusive involvement of all health-care providers, especially providers of poor rural communities.

## Introduction

The global targets for reducing the Tuberculosis (TB) incidence, prevalence and mortality for 2015 have been outlined by the Stop TB Partnership. These targets are set within the overall context of the Millennium Development Goals (MDGs) and are that the global TB incidence rate should be falling by 2015 and that TB prevalence and death rates should be halved by 2015 compared with their levels in 1990 [1], [2].

Due to numerous challenges in measuring incidence, prevalence and mortality, the World Health Organization (WHO) Task Force on TB Impact Measurement has developed a standard framework which outlines the related analyses and tools for this purpose. The major recommendation of this Task Force is that all countries should strengthen their routine surveillance systems (TB-specific recording and reporting systems and/or general health information systems) and ensure that all TB cases are captured by this system [3].

India is one of the high TB burden countries accounting for one fifth of the global incidence of TB and tops the list of 22 high TB burden countries [4]. The only available source of TB patient related information is from the Government of India's Revised National TB Control Programme (RNTCP) which uses standardized recording and reporting systems spread throughout the country for systematically collecting, analyzing and disseminating data. This recording and reporting system is in alignment with the WHO recommended standard recording and reporting system for National TB Programmes and captures information on TB patients initiated on treatment using the drugs and regimens prescribed by RNTCP [5], [6].

TB care in India is provided by both public and non-public sector health facilities [7]. Patients from the public sector are usually managed within the programmatic setting as specified by RNTCP guidelines and are captured by the RNTCP based TB notification system in India. While RNTCP has made concerted efforts to involve non-public health providers in promoting TB care, it is believed that many patients continue to seek treatment from providers outside programme settings [8], [9] and therefore go unreported under the existing TB notification systems [6]. However, evidence from published literature neither provides a reliable estimate nor a proportion for such TB patients who seek care from the non-public health providers.

A community based study was undertaken to estimate the number of self reported TB patients who are currently on TB treatment, the proportion that are accessing TB treatment outside the programmatic setting and their basic socio demographic characteristics.

## Methods

### Study Setting

The Global Fund Round 9 India TB project (IDA-910-G17-T) seeks to increase civil society's support to the national TB programme in India and to engage communities and community based care providers in 374 out of 650 districts across 21 of the 35 states and union territories in the country. These 374 districts were selected based on low TB case detection or because of limited access of populations to health services, In 2011, a baseline survey of knowledge, attitudes and practices of the community to TB was conducted in a representative sample of 30 of the 374 districts to provide pre-project implementation information to inform impact assessment; full results of this baseline survey are under analysis and will be reported separately. A limited dataset collected during this survey from patients undergoing TB treatment was used for this analysis.

### Study design, sample size, sampling and study population

We used a cross-sectional study design. In the absence of reliable estimates, we assumed that 30% of the TB cases in the community are being treated outside RNTCP. A sample size of 710 TB patients was needed to estimate the proportion treated outside RNTCP with a precision of ±5%, considering a 10% non-response rate and with a design effect of 2 to account for cluster sampling. The estimated population prevalence of TB in India is 249 TB cases per 100,000 population [4] and we assumed that 90% of the cases will be on TB treatment. A population of at least 300,000 was required to identify the required number of TB patients for the study.

**Figure 1:**
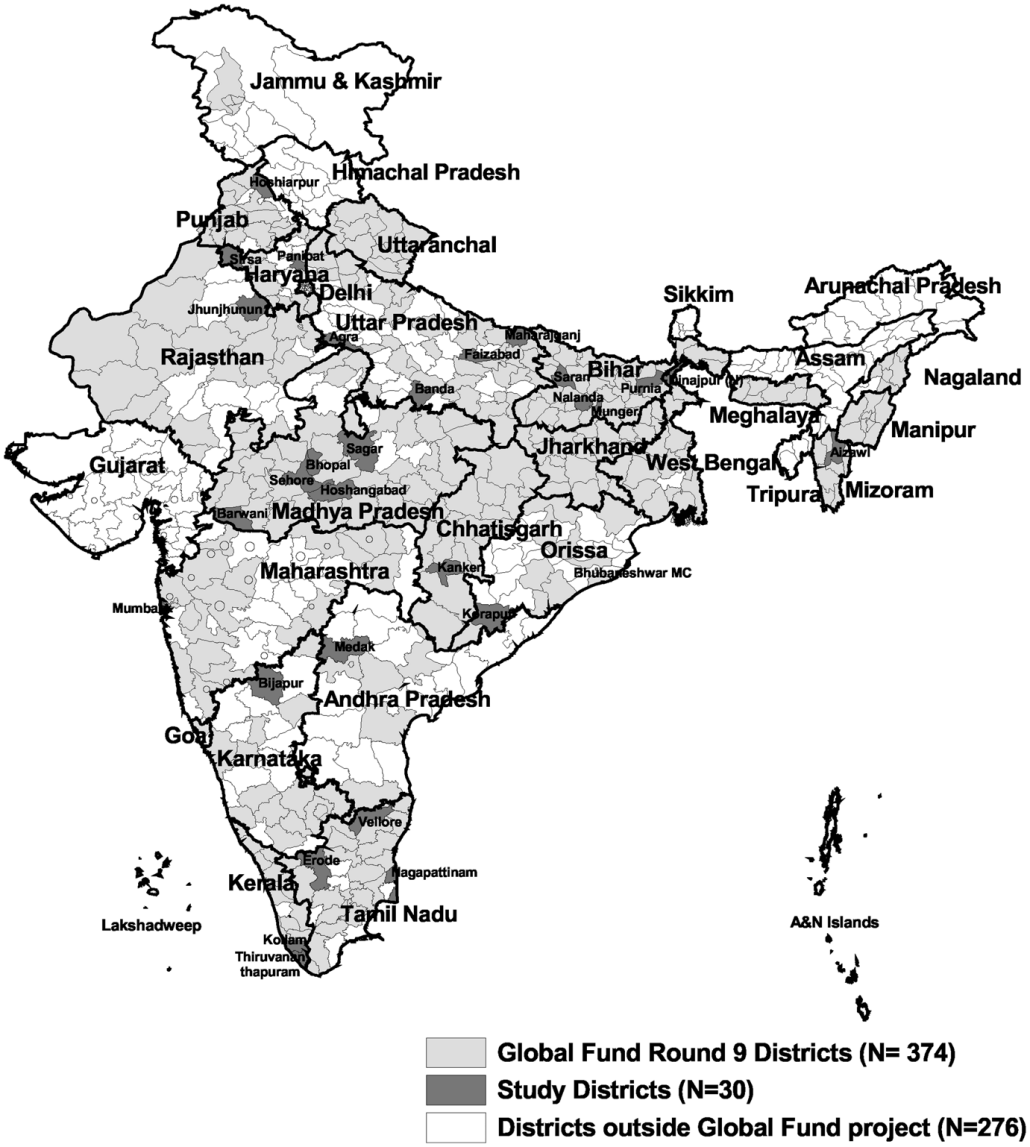
State and district map of India showing the Global Fund Round 9 India TB project districts, and the 30 districts that were selected for the survey.

Thirty districts out of the 374 global fund project districts (**Figure 1**) were selected by a stratified cluster sampling technique. Districts were initially stratified into the 4 RNTCP zones (north, south, east and west) of the country. The number of districts in each zone was selected in proportion to the distribution of the 374 districts in the respective zones of the country and the required number of districts in each zone was selected by population proportionate to size sampling. (**Table 1**).

**Table 1:** Total number of districts selected zone wise from the Global Fund Round 9 Project districts for the cross-sectional community based survey, India, 2011.

Zones	Total number of Districts under the Project	No. of districts to be selected
North Zone	89	7
South Zone	60	6
East Zone	120	9
West Zone	105	8
**Grand Total**	**374**	**30**

From each of these districts, the population was divided into urban and rural primary sampling units of approximately 250 households (the approximate population in each household is 4 and the approximate size of the primary sampling unit is 1000 population), based on the data available from the country's 2001 census. Ten primary sampling units were selected randomly (using the random numbers generated at www.random.org) in each district from the urban and rural primary sampling units in proportion to the districts' estimated urban and rural population.

### Study investigators, data collection, study instrument and study variables

The study was implemented by The Union, South-East Asia Regional Office with assistance from field investigators of the social research organization GfK MODE. The trained field investigators visited the preselected primary sampling units during the months of January to March, 2011 and conducted a household line listing. During this line listing process, TB patients were identified by interviewing heads of the households or other available household members to know whether any current household member was known to be suffering from TB (or an equivalent local term referring to TB). A current household member was defined as a person who is alive and has stayed in the household for at least 6 months prior to this survey.

A semi-structured questionnaire was used to collect information from these identified TB patients (their guardian was selected in case the TB patient was aged less than 18 years). This semi-structured questionnaire included data on age, sex, total current monthly household income from all sources (in Indian rupees), literacy status (an illiterate was considered as a person who cannot read and write in any language), source of TB diagnosis (whether it is government or non-government health facility), site of disease (pulmonary or extra-pulmonary), whether treated for TB in the past and their source of TB treatment. Given the large, decentralised nature of data collection, based on the experiences during pre-testing of the study methodology, the questionnaire was simplified in a manner in which the patients could understand and respond reliably.

We defined operationally the source of TB treatment to be from ‘DOTS/RNTCP’ if patients stated that the drugs they consumed were provided free of cost (as treatment under RNTCP is provided free of cost), by the government health facilities or non-government health facilities, taken thrice weekly from patient wise boxes, and/or the drugs were consumed in the presence of a health worker. Additional information was sought to determine if the patient had an identity card provided by RNTCP. In the absence of this information, or if the drugs were being consumed contrary to this procedure, we defined the patient as taking treatment from ‘outside DOTS/RNTCP’ sources.

### Data entry and analysis

Data collected from the field by the investigators were entered into a pre-structured format in Fox Pro (Version 2.6), cross verified for consistency and were analyzed using Epi-data (version 2.2.1) and Stata (Version 10). Variables were summarized by proportions and 95% confidence intervals (95% CI) were calculated using cluster analysis to account for cluster sampling methodology. Differences between sub-groups were measured by odds ratios (OR) with 95% confidence intervals. All patient variables included in our study were known from previous studies to confound each other. Hence, we have done unconditional logistic regression for calculating the adjusted odds ratio (aOR) for identifying the statistically significant patient characteristics that were associated with accessing treatment from ‘outside DOTS/RNTCP’. A *p*-value less than 0.05 were taken to be statistically significant.

### Ethical Considerations

The study protocol was approved by the Ethics Advisory Group of The Union. In addition, as this is an approved activity under the Global Fund Round 9 project, Central TB Division, Ministry of Health and Family Welfare, Government of India provided consent to this study. Prior to conducting the survey, permission was also sought from the community heads/representatives of the primary sampling units in each district. Written consent was also sought from the heads of households and individual TB patients.

## Results

Of the estimated 75,000 households, 73,249 households (97.6%) were visited during the survey. There was a total of 371,174 household members of whom 761 reported that they were on TB treatment [approximates to 205 TB patients per 100,000 populations; (95% CI: 146–260 TB patients per 100,000 population)] during the household listing process. However, 152 (20%) of these patients could not be interviewed either because, written consent was not given or the head of the household and/or the patients could not be contacted on two attempts on successive days during the survey. Data were collected from 609 (80%) TB patients. There were no statistically significant Zonal and urban-rural differences between the proportion of patients interviewed and those not interviewed.

### The characteristics of TB patients interviewed

The characteristics of the 609 interviewed TB patients are shown in **Table 2**. More than half (64%) of the patients were males. Nearly three fourths (73%) of the patients were in the age group 25–54 years. A large proportion (43%) was illiterate (inability to read and write in any language). Almost eighty percent of patients were from households with a current total household income less than Indian Rupees (INR) 4000 per month from all sources [1 United States Dollar (USD) = 45 INR]. Three fourths (77%) were new TB cases (first episode of tuberculosis). Overall, of the 609 TB patients, 331 [54% (95% CI 42–66%)] were determined to be taking treatment ‘under DOTS/RNTCP’ either from government or non-government health centers and the remaining 278 [46% (95% CI 34–57%)] were on treatment from ‘outside DOTS/RNTCP’ sources.

**Table 2:** Characteristics of self reported TB patients in a community based survey in India, 2011 (n = 609).

Characteristics	N	%	(95% CI)
**Sex**			
Female	220	36.1	(29.9–42.3)
Male	389	63.9	(57.7–70.1)
**Age Group (in years)**			
<15	13	2.1	(0.9–3.3)
≥15 to <25	99	16.3	(12.7–19.7)
≥25 to <35	101	16.6	(13.8–19.2)
≥35 to <45	134	22.0	(17.9–26.9)
≥45 to <55	107	17.6	(13.6–21.5)
≥ to <65	95	15.6	(12.3–18.8)
≥65	60	9.9	(6.9–12.7)
**Literacy status**			
illiterate	264	43.3	(35.0–51.6)
literate	345	56.7	(48.4–65.0)
**Current monthly household income from all sources (in INR)** *			
<2000	212	34.8	(25.9–43.6)
2000–4000	270	44.3	(38.1–50.5)
4001–8000	64	10.5	(6.1–14.8)
8001–10,000	22	3.6	(1.3–5.9)
>10,000	11	1.8	(0.1–3.4)
Don't Know	30	4.9	(1.1–8.7)
**Residence**			
Rural	468	76.8	(64.1–89.5)
Urban	128	21.0	(8.6–33.4)
Unknown	13	2.1	(0.3–3.9)
**Type of TB**			
New	470	77.2	(69.6–84.7)
Previously treated	139	22.8	(15.3–30.4)
**Source of TB diagnosis**			
Government health facility	366	60.1	(46.3–73.8)
Non-Government health facility	236	38.8	(25.3–52.1)
others/unknown	7	1.1	(0.3–1.9)
**TB Site**			
Pulmonary	573	94.1	(91.5–96.6)
Extra-pulmonary	28	4.6	(2.8–6.3)
Unknown	8	1.3	(0.0–2.6)
**Source of TB treatment**			
Government health centres, free of cost under DOTS/RNTCP	310	50.9	(38.4–63.4)
Non-government health centres, free of cost under DOTS/RNTCP	21	3.4	(1.6–5.2)
Government health centres, with payment for medicines (outside DOTS/RNTCP)	36	5.9	(3.1–8.6)
Non government health centres, with payment for medicines (outside DOTS/RNTCP)	218	35.8	(24.5–47.0)
Other sources-non allopathic medicines(outside DOTS/RNTCP)	24	3.9	(1.8–6.0)

*1 United States Dollar = ∼45 Indian National Rupees.

### Characteristics of TB patients in relation to source of TB treatment

The bivariate analysis showing characteristics of TB patients in relation to their source of TB treatment are shown in **Table 3**. While a large proportion of TB patients accessing treatment ‘outside DOTS/RNTCP’ were illiterate (48%) and from rural areas (86%), the characteristics that were statistically significant when compared to those accessing treatment ‘under DOTS/RNTCP’ were current household income (≤INR 4000), setting (rural) and the source of diagnosis (non-government health facility). Some crossover between diagnosis and treatment was observed; 14% of those diagnosed in the private sector were treated ‘under DOTS/RNTCP’, while 30% diagnosed at the government health facility sought treatment ‘outside DOTS/RNTCP’. In addition, not all patients treated from the government health facilities were treated ‘under DOTS/RNTCP’ and ∼6% were treated ‘outside DOTS/RNTCP’—an unusual observation as nationwide public health facilities do not independently procure first-line anti-TB drugs.

**Table 3:** Characteristics of self Reported TB patients (n = 609) in relation to their source of TB treatment in a Community based Survey in India, 2011 (Bi-variate analysis).

Characteristics	Outside DOTS/RNTCPN (%)		Under DOTS/RNTCP		Odds ratio(95% CI)
			N	(%)	
**Sex**					
Female	108	(39)	112	(34)	1.24 (0.9–1.7)
Male	170	(61)	219	(66)	Referent
**Age Group (in years**)					
<25	54	(19)	58	(18)	1.16 (0.5–1.3)
25–54 years	152	(55)	190	(57)	Referent
≥55	72	(26)	83	(25)	1.08 (0. 7–1.7)
**Literacy status**					
Illiterate	133	(48)	131	(40)	1.40 (0.8 –2.3)
Literate	145	(52)	200	(60)	Referent
**Current monthly household income (in INR)** *					
≤4000	237	(85)	245	(74)	1.96 (1.1–3.4)**
>4000	32	(12)	65	(20)	Referent
Unknown	9	(3)	21	(6)	
**Setting**					
Rural	239	(86)	229	(69)	2.66 (1.1–6.3)**
Urban	36	(13)	92	(28)	Referent
Unknown	3	(1)	10	(3)	
**Type of TB**					
New	219	(79)	251	(76)	1.18 (0.5–2.5)
previously treated	59	(21)	80	(24)	Referent
**Body site affected by TB**					
Pulmonary	263	(95)	310	(94)	1.79 (0.6–5.2)
Extra-pulmonary	9	(3)	19	(6)	Referent
Unknown	6	(2)	2	(1)	
**Diagnosis Source**					
non-Government health facility	191	(69)	45	(14)	14.47 (8.6–24.4)**
Government health facility	83	(30)	283	(85)	Referent
others (including unknown)	4	(1)	3	(1)	

*1 United States Dollar = ∼45 Indian Rupees (INR).

**Statistically significant.

Based on multivariate analysis (**Table 4**) patients who were accessing treatment from ‘outside DOTS/RNTCP’ were more likely to be patients from rural areas [adjusted odds ratio (aOR) 2.5, 95% CI 1.2–5.3]. The characteristic most strongly associated with treatment ‘outside DOTS/RNTCP’ was TB diagnosed in a non-government health facility (aOR14.0, 95% CI 7.9–24.9).

**Table 4:** Multivariate analysis for characteristics associated with patients accessing TB treatment ‘outside DOTS/RNTCP’ in a community based survey, India, 2011 (N = 555).

Characteristics	Adjusted odds ratio	(95% CI)	P- Value
**Sex**			
Male	referent		
Female	1.24	(0.8–1.9)	0.299
**Age Group**			
25–54 years	referent		
<25	1.08	(0.6–2.1)	0.787
> = 55	0.92	(0.5–1.7)	0.782
**Literacy status**			
literate	referent		
Illiterate	1.26	(0.7–2.2)	0.380
**Current monthly household income (in INR)** *			
>4000	referent		
≤4000	1.81	(0.9–3.7)	0.100
**Setting**			
Urban	referent		
Rural	2.48	(1.2–5.3)	0.021**
**Type of TB**			
previously treated	referent		
New	0.73	(0.4–1.3)	0.292
**Body site affected by TB**			
Extra-pulmonary	referent		
Pulmonary	2.94	(0.9–9.9)	0.079
**Diagnosis Source**			
Government health facility	referent		
Non-Government health facility	14.03	(7.9–24.9)	<0.001**

*1 United States Dollar = ∼45 Indian Rupees (INR).

**Statistically significant.

## Discussion

This is one of the few community-based surveys in India providing information on the overall prevalence of patients on TB treatment (by self-report) and their source of diagnosis and treatment. This population-based survey of more than half the districts in the country found 205 self-reported TB patients per 100,000 populations. This finding highlights that TB remains a disease of public health importance in India, and that the disease burden remains high after more than a decade of intensive TB control efforts led by RNTCP. In addition, they also help to identify the profile of patients who are not accessing TB treatment services under RNTCP.

This finding is consistent with that from other surveys of TB prevalence and self-reported TB prevalence from India, though nationally-represented prevalence data are not available [4]. Data from the 3^rd^ National Health and Family Survey (NHFS) had shown that prevalence of medically treated TB was 418 per 100,000 usual household residents, with higher prevalence in men. NHFS however, did not refer only to patients being currently treated for tuberculosis as has been done in our survey [10].

What are the implications of this study for RNTCP? First, the current TB notification system requires expansion to reach patients diagnosed and treated outside direct RNTCP services. RNTCP is moving towards ‘universal access’ to TB diagnosis and treatment under RNTCP and aims to detect and treat at least 90% of the estimated TB cases in the community [11]. In the absence of nationally representative surveys, the TB disease burden has to be estimated indirectly (using the ‘onion model’) from the data on TB notification as outlined by the WHO Task Force on TB impact measurement [3]. As mentioned earlier, the TB notification system in India is based on TB cases accessing treatment under the RNTCP and it is known that a large proportion of TB patients access treatment outside the RNTCP. In this scenario, one of the key pieces of information required for the indirect estimation is an answer to the question “What fraction of cases is missed in TB notification data”? This study, by providing information that 46% (95% CI 34–57%) of TB patients may not be notified under the programme, provides data for estimating the burden of TB by this indirect method as outlined by the WHO Task Force on TB Impact Measurement [3]. In order to make the TB notification system in India complete, mechanisms have to be initiated in India to capture these TB cases that are accessing treatment ‘outside RNTCP’ by expanding the scope of the current TB notification system.

Second, TB has been and remains a disease that largely afflicts the impoverished, and that needs to be incorporated into TB programme planning. Universally, the poor and socially vulnerable groups are at higher risk for TB disease and death [12]. Although the national programme in India is designed to benefit poor and vulnerable communities in the country, data from this study show that large proportions of patients who are accessing treatment ‘outside DOTS/RNTCP’ are illiterate, live in very low income households, in rural areas and have to pay for their treatment. The current levels of income in households of patients who are on treatment are likely to be lower than their past and regular incomes, because of inability to work, or return to full work. This has important implications for TB control and the alleviation (or exacerbation) of poverty in the country. Reasons for patients seeking care from outside the national programme are many, and include poor knowledge about the disease and the services available through the national programme [13], [14]; they also include barriers such as convenience of the services, confidentiality and a desire for personalized care [15].

Third, crossover of patients after diagnosis at the stage of seeking treatment or even during treatment, from one type of healthcare provider to another was observed in our study. A study on care seeking behavior in South India showed that the RNTCP has had an impact in the community with regard to the availability and accessibility of TB services in government health facilities. However relatively large numbers of the chest symptomatic patients had subsequently shifted to the non-Government health facilities prompting the authors to recommend urgent measures to make government facilities more patient friendly [16]. Another study in Delhi during the early phase of RNTCP implementation had shown that health workers screened TB patients to assess whether the patients would adhere to treatment. In this process, patients mainly those who were in absolute poverty, socially marginalized, itinerant labourers, poorly integrated in the city, were not put on treatment regimens as recommended under RNTCP as the health workers felt that these patients would not adhere to treatment [17]. The large advocacy, communication and social mobilisation project coordinated by civil society through the Global Fund Round 9 India TB grant has the potential to address these barriers and complement the national programme's efforts in reaching poor and vulnerable communities.

Fourth, TB patients who were diagnosed in the non-government health facilities are more likely to be treated outside the programme setting, and this may not be in accordance with the patient management outlined in International Standards of TB care (ISTC) [8], [18]. The number of such non-governmental health facilities in India run into hundreds of thousands. It is estimated that over 80% of all health care in the country is accessed from the non government sector [19], with less than 45% of the inpatient care sought from the government (public) health facilities [20]. Data from the 60^th^ Round of the National Sample Survey Organisation of India, corresponding to the year 2004, had shown that younger age group, women, people with higher level of education and economic status were more likely to avail treatment at non-government sector facilities [21]. Tremendous efforts have been made by the TB programme to reach out to the vast non-government sector health facilities through various innovative mechanisms using public private mix approaches and by advocacy through various medical professional associations. These efforts, however, appear to be inadequate given the health care system in the country [22], [23]. The responsibility for participating in organized TB control efforts also rests with all health care providers who manage TB patients as per the ISTC, and this message needs to be communicated to all the health care providers in the country as many may not be aware [24], [25].

### Limitations

While we believe that the findings are valid, there are some limitations to the study. **First**, these data are not nationally representative but representative of the 374 Global Fund Round 9 ACSM intervention districts. As mentioned previously under study setting, these districts were selected for the project interventions by RNTCP based on their relatively poor programme performance. The situation may or may not be the same in other 276 districts of the country. **Second**, the study identified TB patients based on a door to door household survey and by enquiring about TB disease status (Self reported). This methodology has its limitations in that only diagnosed TB patients who voluntarily disclose their disease and treatment status will be captured. If the patients are not diagnosed in the community, or if they do not disclose their disease status voluntarily due to reasons such as stigma, then this survey would have missed such cases. **Third**, we were not able to cross check the accuracy of the TB disease diagnosis and the appropriateness of treatment as the clinical records that were available with many patients (∼40%) were incomplete. If for any reason, the diagnosis of TB disease was inaccurate or if the therapy was inappropriate, then our study findings may not have provided the correct picture of the patients on TB treatment. **Fourth**, we were not able to interview nearly 20% of the TB patients identified in this survey due to certain operational and ethical reasons as mentioned above. The only two variables by which we could assess whether the patients interviewed were similar to the patients not interviewed were the zone and the urban/rural status of the patients. If there were differences in other variables between those included and not included, then this has to potential to change the study results. These are usual limitations of any community based surveys.

### Conclusion and recommendations

India has declared the intent to achieve by 2017 ‘universal access’ to TB diagnosis and treatment for all TB cases in the community, and to extend RNTCP services to patients diagnosed and treated in the private sector [11] Nearly half of all patients treated for TB in these 30 districts are treated ‘outside DOTS/RNTCP’ sources and many not be notified. The study highlights the need for future research, programme policies and activities on 1) Reviewing and revising the scope of the TB notification system, 2) Further strengthening and monitoring of health care delivery systems especially to the rural communities with periodic assessment of reach and utilisation of the TB services 3). Advocacy, communication and social mobilisation activities focused at rural communities with low household incomes and 4) Inclusive involvement of all care providers, especially non government providers of poor rural communities.
